# Compensatory Base Changes in ITS2 Secondary Structure Alignment, Modelling, and Molecular Phylogeny: An Integrated Approach to Improve Species Delimitation in *Tulasnella* (Basidiomycota)

**DOI:** 10.3390/jof9090894

**Published:** 2023-08-31

**Authors:** Yuliana Jiménez-Gaona, Oscar Vivanco-Galván, Darío Cruz, Angelo Armijos-Carrión, Juan Pablo Suárez

**Affiliations:** 1 Departamento de Química, Universidad Técnica Particular de Loja (UTPL), San Cayetano Alto s/n, Loja 1101608, Ecuador; 2 Departamento de Ciencias Biológicas, Universidad Técnica Particular de Loja (UTPL), San Cayetano Alto s/n, Loja 1101608, Ecuador; oavivanco@utpl.edu.ec (O.V.-G.); djcruz@utpl.edu.ec (D.C.); jpsuarez@utpl.edu.ec (J.P.S.); 3 Department of Biology, Memorial University of Newfoundland, St. John’s, NL A1B 3X9, Canada; aarmijoscarr@mun.ca

**Keywords:** alignment-based, alignment-free, compensatory base changes, ITS2, secondary structure, species delimitation

## Abstract

Background: The delimitation of species of *Tulasnella* has been extensively studied, mainly at the morphological (sexual and asexual states) and molecular levels—showing ambiguity between them. An integrative species concept that includes characteristics such as molecular, ecology, morphology, and other information is crucial for species delimitation in complex groups such as *Tulasnella*. Objectives: The aim of this study is to test evolutionary relationships using a combination of alignment-based and alignment-free distance matrices as an alternative molecular tool to traditional methods, and to consider the secondary structures and CBCs from ITS2 (internal transcribed spacer) sequences for species delimitation in *Tulasnella*. Methodology: Three phylogenetic approaches were plotted: (i) alignment-based, (ii) alignment-free, and (iii) a combination of both distance matrices using the DISTATIS and pvclust libraries from an R package. Finally, the secondary structure consensus was modeled by Mfold, and a CBC analysis was obtained to complement the species delimitation using 4Sale. Results and Conclusions: The phylogenetic tree results showed delimited monophyletic clades in *Tulasnella* spp., where all 142 *Tulasnella* sequences were divided into two main clades A and B and assigned to seven species (*T. asymmetrica*, *T. andina*, *T. eichleriana ECU6*, *T. eichleriana ECU4 T. pinicola*, *T. violea*), supported by bootstrap values from 72% to 100%. From the 2D secondary structure alignment, three types of consensus models with helices and loops were obtained. Thus, *T. albida* belongs to type I; *T. eichleriana*, *T. tomaculum*, and *T. violea* belong to type II; and *T. asymmetrica*, *T. andina*, *T. pinicola*, and *T.* spp. (GER) belong to type III; each type contains four to six domains, with nine CBCs among these that corroborate different species.

## 1. Introduction

Species delimitation is an uncertain issue, particularly in fungi. Several criteria are available, and the selection of the better alternative depends on the characteristics of each taxonomic group [1]. During the last two decades, and thanks to the advent of high-throughput sequencing technologies, many efforts have approached species delimitation at the level of DNA sequencing [2]. Such efforts have led to the development of the field of molecular phylogenetics, which aims to address the concept of species delimitation through phylogeny estimation from sequence data [3,4]. The hypothesis behind molecular phylogenetics is that genomes evolve by the gradual accumulation of mutations. Therefore, the level of difference in the nucleotide sequences between a pair of genomes should indicate how recently those two genomes shared a common ancestor [5].

Up to now, the most accepted approach for species delimitation from sequence information relies on the existence of so-called universal barcodes—for instance, short DNA regions placed in the nuclear, mitochondrial, and/or chloroplast DNA that evolve at a rather constant rate. DNA barcoding [6] has provided valuable evidence for resolving misidentified species and identifying cryptic species within morphologically complex groups such as *Tulasnella* [7].

In addition, species delimitation from barcode sequences has traditionally relied on the generation of multiple sequence alignments (MSA) [8]. The interpretation of MSA assumes that homologous positions across species occur in the same order relative to one another (homology), which is not always true due to the recombination, rearrangement, and lateral genetic transfers that all occur naturally in genes and genomes [9,10]. Therefore, in many species, the positional hypothesis of homology generated by MSA is incomplete or incorrect, which has the effect of diffusing the phylogenetic signal of barcodes and hampering species delimitation—especially when approached by a single-locus strategy.

Some alternatives to MSA in phylogenetic inference were introduced to improve species delimitation from sequence data: the so-called alignment-free approach, which computes pairwise similarity from sequences [11], and the distance integration method. In this sense, there are two popular methods for performing such data integration steps: (i) concatenation (the supermatrix method) and (ii) consensus (the supertree method) [8]. 

Despite the clear benefits of using a multi-locus strategy instead of a single-locus approach, both have the handicap of relying on MSA. Generating accurate alignments is challenging, as errors introduced during this step significantly affect phylogenetic reconstruction and species delimitation. However, computational methods that implement data integration schemes are known to offer improved solutions [9,10]. Abeysundera et al. [11] reported an accurate alternative method for combining distance matrices (and not gene trees) from multiple single genes on identical taxon sets to obtain a single representative distance matrix, and to derive a species tree. 

A number of successful approaches have been based on deriving distance matrices from ITS2 structural features that are shared among all eukaryotes [12,13,14,15,16,17].

Consequently, the secondary structure of ITS2 provides additional information for resolving the challenges of identifying closely related fungal species [10,11,12]. The ITS2 secondary structure is being increasingly used in the field of phylogenetics due to several reasons: (i) Secondary structure analysis extends the taxonomic applicability of this marker [13]; (ii) the results from secondary structure analysis can improve the phylogenetic resolution obtained from the primary sequence [18]; and (iii) the structural data can provide new information via compensatory base change (CBC) analysis, and thus provide a tool for species delimitation [19]. In fact, the secondary structure is a prerequisite for all conclusions derived from the phylogenetic analysis.

This has led to active research in molecular phylogenetics on alignment-free methods for reconstructing phylogenies. These methods integrate structural alignment-based and alignment-free features into distance matrices, which are then used to infer phylogenetic trees with good resolution at the species level [20,21].

The ITS2 secondary structure provides features such as the so called compensatory base changes (CBCs, nucleotide substitutional events), suggested as additional evidence for the distinguishing of some closely related species [22,23]. Wolf et al. [23] mentioned that if there is a CBC, then the samples are two different species, at a probability of ~93%; if there is not a CBC, then the samples are the same species, at a probability of ~76%. This approach includes searching for co-variations (i.e., CBCs—e.g., C-G → U-A—and hemi-CBCs, e.g., A → U), common structural motifs, and indels by comparing every single nucleotide/base pair in homologous ITS2 regions of the predicted structures [24,25].

Many studies have used structural information to estimate phylogenies. Agüero-Chapin et al. [26] reported a pure alignment-free strategy for estimating phylogenies from ITS2 sequences, which was used to complement the morphological characterization of some endophytic fungi (*Neurospora* and *Gelasinospora* genus) of living plants that had been challenging to place taxonomically. Additionally, in [27,28], several alignment-free methods for phylogeny estimation were published. Furthermore, Cheng et al. [29] created a web server to facilitate the use of various alignment-free approaches for alignment-free phylogeny estimation.

Despite the vast number of available methods for phylogeny estimation (single-locus, multi-locus, structural feature-based, and alignment-free), the benefits of integrating different approaches have been poorly explored; this is probably due to the limited availability of algorithms to combine distance matrices derived from different metrics [9,10].

In previous studies, Abeysundera et al. [11] combined alignment-free distances based on spectral covariance (SpCov) [30]. Inspired by the work of Agüero-Chapin et al. [7] and Abeysundera et al. [30], we hypothesized that integrating the alignment-free (structural features) and alignment-based distances of ITS2 sequences into a single matrix, including the identification of CBCs between the 2D structural helices, would improve species recognition. To test our hypothesis, we used a published dataset of the complex fungal group *Tulasnella (Basidiomycota)*, where species delimitation based on molecular and taxonomical data is usually ambiguous.

## 2. Materials and Methods

### 2.1. Datasets

One fungal dataset involving 142 sequences of *Tulasnella* spp. was selected for this analysis. Most of the delimited species had already been discussed on the morpho-molecular level by Cruz et al. 2014 [7]. Sequences including the complete ribosomal nrITS-5.8S region were downloaded from the Genbank database National Center for Biotechnology Information (NCBI; http://www.ncbi.nlm.nih.gov/, accessed on 1 February 2022) [31]. The flanking 28S and 5.8S regions were excluded by the ITSx v1.0.11 tool [17,32]. The molecular data used to reconstruct the phylogenies consisted of only ITS2 sequences. Finally, one outgroup sequence belonging to *Puccinia boraniae* (AY348716) was added to the dataset using the Mafft-add tool [33]. This outgroup was selected because it does not share close characters with *Tulasnella* spp. and allowed us to strongly mark the formation of phylogenetic clades in this highly variable group.

**Figure 1:**
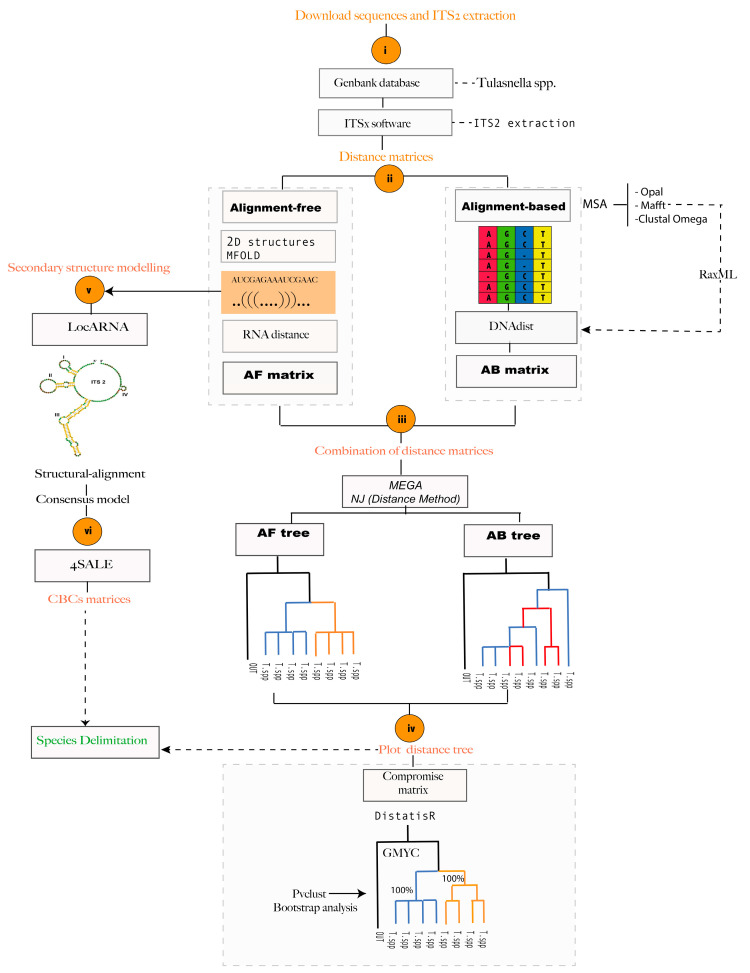
Flowchart summary to delimit species boundaries in *Tulasnella* genus. (**i**) Download Tulasnella sequences with total region ITS-5.8S from Genbank database [31]; ITS2 region extraction with ITSx v1.0.11 software [17,32]; (**ii**) Obtain two distance matrices from Alignment-based and Alignment-free; (**iii**) Combine the distance matrices by Distatist [9], (**iv**) Plot the resulting phylogenetic tree using the pvclust library (R package) [34]; (**v**) Secondary structure modelling and; (**vi**) Define the CBCs as additional molecular markers to delimit *Tulasnella* species.

Figure 1 shows the workflow summary proposed to delimit *Tulasnella* species in this work. 

### 2.2. Phylogenetic Reconstruction

The phylogenetics strategy for the distance matrices followed these next steps:

(i) Alignment-free (AF) matrices from ITS2 secondary structure. 

First, the 2D structures were inferred by a MFE (free energy minimization) parameter from Mfold [35]. Then, the distance matrix from the predicted structures was obtained with RNA distance (Vienna Package v2.0) [36,37], which was used as the input for constructing a first Neighbor Joining (NJ) phylogenetic tree with MEGA6.0 [38] to estimate an AF-based phylogenetic tree. 

(ii) Alignment-based (AB) matrices from Opal MSA. 

Afterwards, three multiple sequence alignment (MSA) methods were selected for inferring homology: Clustal Omega v1.2.1 [39], MAFFT v7 http://mafft.cbrc.jp/alignment/software, accessed on 2 February 2022 [40], and OPAL v2.1.3 http:/opal.cs.arizona.edu, accessed on 2 February 2022 [41]. These tools were chosen due to their effectiveness in dealing with high sequence heterogeneity, such as is the case in the ITS2 region. The MSA accuracy was evaluated through the calculation of the Maximum likelihood (ML) optimization score by the RaxML HPC2 web tool (https://cme.h-its.org/exelixis/web/software/raxml/index.html, accessed on 2 February 2022) [42], which provide several statistics to assess the best MSA technique (aligns more consistently with fewer ambiguous regions) through the highest negative value (the negative log-likelihood, -Lnl). 

The AB tree was developed based on Opal MSA with bootstrap = 3000 repetitions, which obtained the highest negative score (-InL of −4755.596439) compared to the other two methods: Mafft (InL = −3760.694766) and Clustal Omega (InL = −3766.491609).

Then, DNAdist (https://evolution.genetics.washington.edu/phylip/doc/dnadist.html, accessed on 2 February 2022) was used to obtain the distance matrix from Opal MSA, which was used as the input for constructing a second NJ phylogenetic tree with MEGA6.0 [40] to obtain an AB phylogenetic tree. 

(iii) AB and AF distance matrices combination.

To delimit species boundaries by phylogenetic analyses, the two resulting matrices from AB and AF were combined into a single-compromise matrix (*data*) using DistatisR, which evaluates the similarity between distance matrices. The compromise matrix represents the best aggregate of the original matrices. The following parameters were used: 

***test<-distatis (data*** **, *Norm =“MFA”*, *Distance = TRUE*, *RV = TRUE*, *nfact2keep = 2*, *compact = FALSE)***

The compromise matrix was used as the input data to construct the third phylogenetic tree using the pvclust [35] library (R package v1.0), using a bootstrap analysis (*nboot* = 1000), distance measure matrix (*method.dist *= *minkowski*), and single cluster analysis (*method.hclust* = *singl*e). A summary of the procedure to calculate the distance matrix is given in [9,11]. 

(iv) Generalized mixed Yule-coalescent (GMYC) model

Finally, the detection of monophyletic clades was identified in the resulting tree using the GMYC model [43]. They were estimated by running multiple-GMYC (M-GMYC). The support value of a node is defined as the sum of the Akaike weights of the candidate delimitation models in which the node is included:

tree<-read.tree(‘tree.newick’)

mtree<-compute.brtime(tree, method = “multiple”, interval = c(0, 10))

Mtree<-gmyc.heuristic(mtree)

support<-gmyc.support(tree, *p* = 0.95)

The significance of the MGMYC models was evaluated through the likelihood ratio test (LRT), *p*-value < 0.05. 

### 2.3. ITS2 Consensus Secondary Structure Modelling

**Figure 2:**
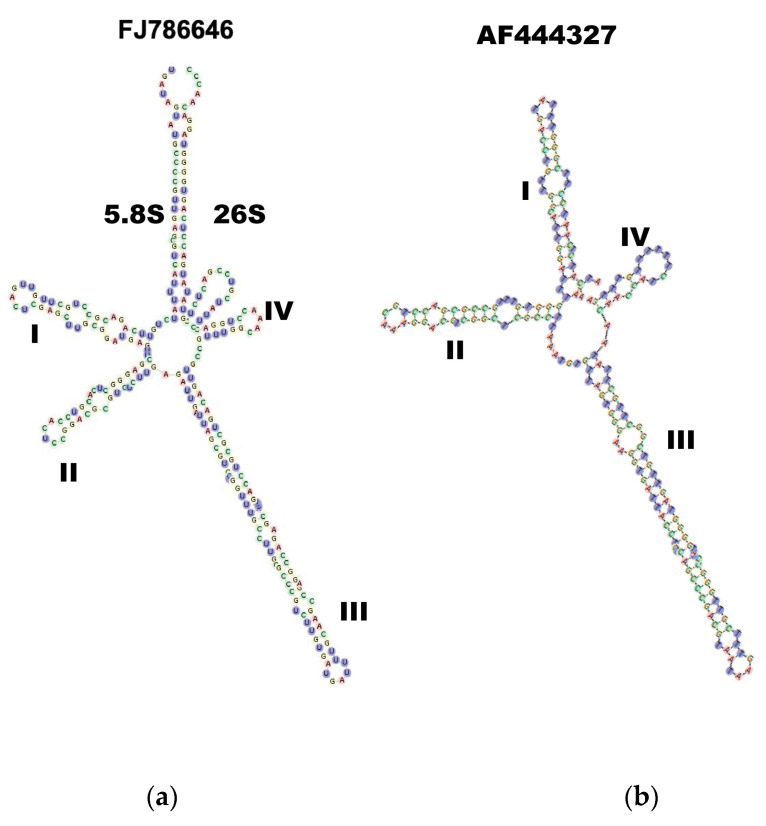
(**a**) *Uncultured Tulasnellaceae* (Genbank FJ786646) secondary structure for the 26 partial ITS-5.8, sequence clone PA195. Helices were numbered I–IV from 5′ to 3′ direction (**b**) Secondary structure of the ITS2 transcript of *Colletotrichum gloeosporioides* (Genbank AF444327).

The ITS2 database http://its2.bioapps.biozentrum.uni-wuerzburg.de/, accessed on 2 February 2022 [44] does not provide the right *Tulasnella* models due to its lack of templates; thus, we used the *Uncultured Tulasnellaceae (*design by Wolf M, collaborator 2010) as a secondary structure comparative model with respect to *Colletotrichum gloeosporioide (*modeled by ITS2 database), which helped as a control for the genus *Tulasnella—*see Figure 2a,b.

**Figure 3:**
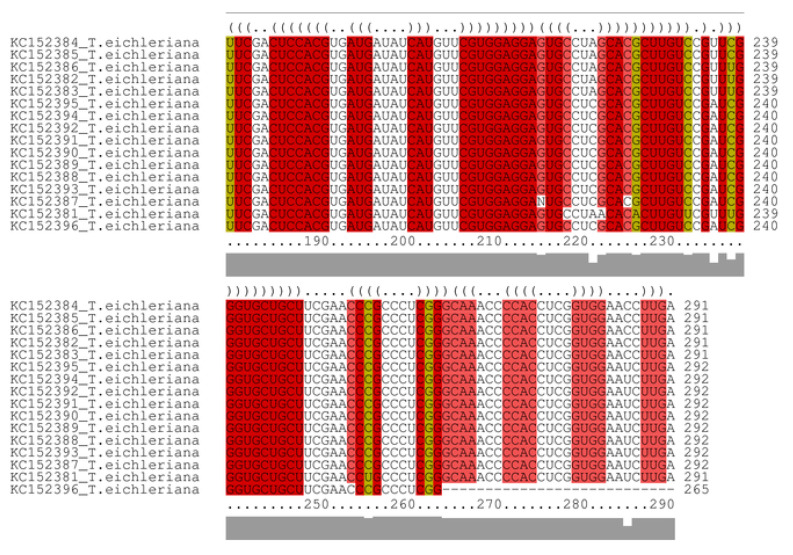
ITS2 sequence structure-based alignment generated by LocaRNA-P. Dot-bracket notation represents unpaired bases and matching parenthesized positions represent paired bases. The red regions indicate structure reliability, the yellow regions represent sequence reliability, and the thin line shows the combined column-reliability.

The secondary structures were individually folded by the Mfold v 3.6 [35] webserver (http://www.unafold.org/mfold/applications/rna-folding-form.php, accessed on 2 February 2022), based on default thermodynamic parameters [45]. Then, these structures were input into LocARNA v2.0 (https://rna.informatik.uni-freiburg.de/LocARNA/Input.jsp;jsessionid=115161C5C9E9AB44CC6D3C81763A1699, accessed on 2 February 2022) [46] software to perform structural-alignment and consensus secondary structure modelling—see Figure 3. 

### 2.4. Compensatory Base Changes (CBCs)

From the secondary-structure alignment, an extra molecular marker named compensatory base changes (CBCs) was considered to delineate the species [22,47]—from which, specific structural features were extracted using the 4SALE tool (http://4sale.bioapps.biozentrum.uni-wuerzburg.de/, accessed on 2 February 2022) [48].

## 3. Results

### 3.1. AB and AF Phylogenetic Reconstruction

**Figure 4:**
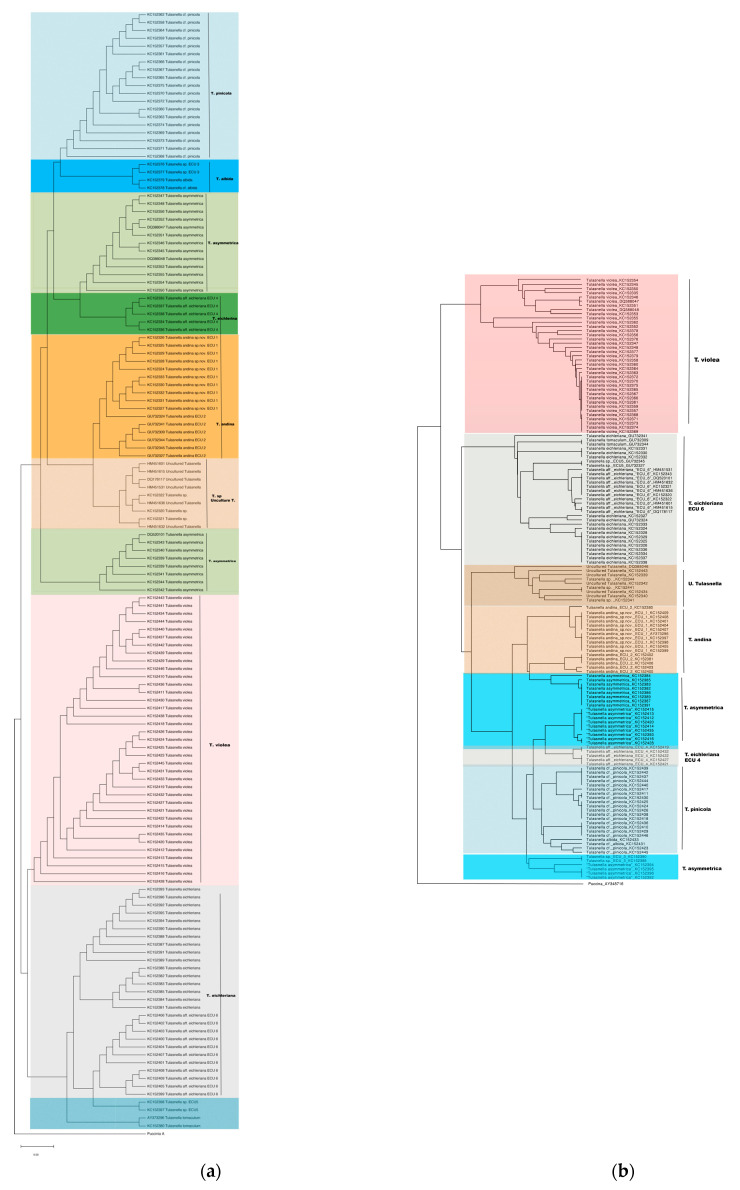
(**a**). AB tree built with Opal [42] and MEGA [38] tools, showing 11 clades, and (**b**). AF tree built with VRNAdistance [37] and MEGA [38] tools, with 9 clades. AB tree matrix carries information about the evolutionary changes in the ITS2 sequences and the AF matrix carries information about changes in their secondary structure.

The phylogenetic trees generated using the NJ-based methods—alignment-based (AB) see Figure 4a and alignment-free (AF) see Figure 4b—showed different monophyletic clades. The relationship between the clades could not be fully resolved.

### 3.2. AB and AF Distance Integration with DistatisR

**Figure 5:**
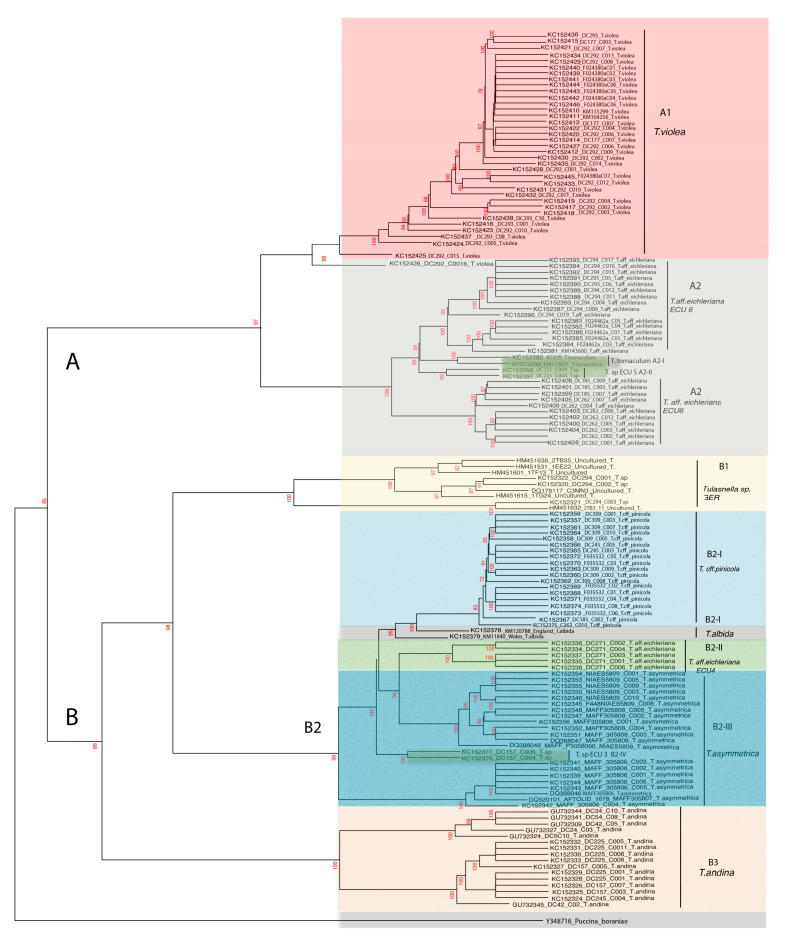
The M-GMYC phylogenetic tree displays the *Tulasnella* species division into different clades and subclades distinguished by colors: *T. violea* (in red), *T. eichleriana* (in grey), *T. tomaculum* (in green), *T.* sp_GER (in yellow), *T. pinicola* (in light blue), *T. albida* (in grey), *T. asymmetrica* (in blue), and *T. andina* (in brown), with two main monophyletic clades (**A**) (**A1**,**A2**) and (**B**) (**B1**,**B2**[**I**–**III**],**B3**). (**A1**,**A2**,**B1**,**B2-I**,**B2-II**,**B2-III**,**B3)** represent monophyletic clades identified by M-GMYC analysis using the R tool (see Table 1).

The distance integration phylogenetic tree obtained from the AB and AF matrix combination is shown in the next phylogenetic tree (Figure 5), where clades were significantly delimited according to their morphology in clade A and B. The clades reflected significant bootstrap values (range from 72% to 100%) between species, and most of the molecular characteristics matched with the results submitted by Cruz et al. [49], who also carried out a morphological analysis (the size and shape of hyphae, basidia, sterigmata, and basidiospores) to complement *Tulasnella* species delimitation. Only two clades did not have complete correspondence (*T. eichleriana*, which includes *T. tomaculum*, *T.* sp_ECU5) and (*T. asymmetrica*, which includes *T.* sp_ECU3), as defined in [49].

As is depicted in Table 1, the LR scores for the M-GMYC were the lowest: *p*-value < 0.05 for the Distance method with respect to the other two. However, AB tree-grouping species of the same genus showed better *p*-values than the AF tree.

### 3.3. Consensus Secondary Structure Modelling for Intra-Specific Differentiation

**Figure 6:**
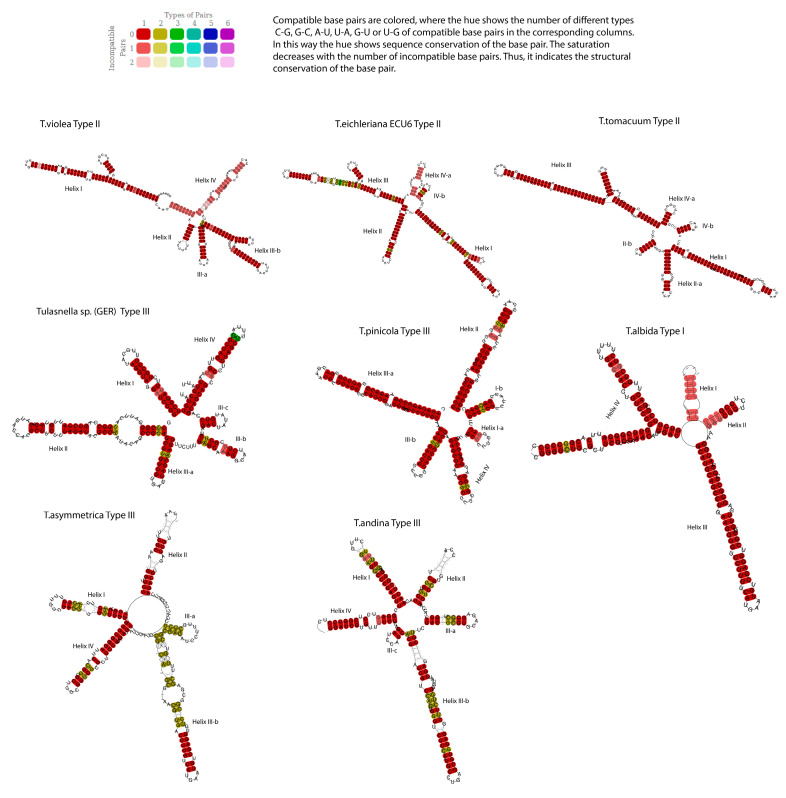
A consensus secondary structures of ITS2 based on minimum free energy (MFE). The colors indicate structural conservation according to the key in the figure, where the homologous regions highlighted in red contain the highest probability of compatibility, with a radiating central and internal loop interconnected with unpaired nucleotides (colorless) in the helices.

Hence, in relation to the previous monophyletic clades and subclades, the ITS2 consensus secondary structure was modeled using structure-based alignment. All predicted secondary structures were arranged to obtain a robust consensus model [50]; it was essential to approximate the phylogenetic and evolutionary relationships between the organisms. The shape and length of each structural helices were manually inspected, and the final consensus models are illustrated in Figure 6.

Figure 6 displays different consensus secondary structure prototypes, revealing three types of models that matched with the subclades shown in Figure 5. Type I consisted of *T. albida*, with the standard model of four domains (helices from I to IV).

Type II included *T. violea*, *T. eichleriana* (*ECU6*), and *T. tomaculum* with a 5-domains, where domain III and IV were divided into two and three subdomains (a–b–c). *T. eichleriana* was divided into original and genetic species; clade A contained the sequences from Europe and was considered as the original species, and clade B contained *T. aff. eicheleriana ECU*, considered genetic species that are different but that resemble each other in their morphology—according to Cruz et al. Thus, the secondary structure consensus type II model was applicable to both groups, and so was considered a conspecific species. Type III included *T.* sp. (GER), *T. pinicola*, *T. asymmetrica*, and *T. andina* with 6-domains, whose helices were divided into two and three subdomains (a–b–c).

### 3.4. CBCs in the ITS2 Secondary Structure

As exemplified for each internal sub-clade A (A1, A2[I–II]) and B (B1, B2[I–IV], B3), typically, the CBCs were equal to 0 between the same species (e.g., A1 -> A1). However, among the external sub-clades (the variants between clades A and B), there was a group of variants with at least one CBC with respect to the remaining groups—with a total of 645 CBCs between all the internal and external subclades (Appendix B). We directly averaged the whole variant CBC matrix (presence/absence) for each species [23,51], as shown in Table 2.

Based on the sequence-structure information, CBC matrices were calculated using the algorithms implemented in 4SALE [48], where different numbers of CBCs were discovered in the conserved regions (helices II and III) between two organisms, which were correlated with morphological incompatibility.

In summary, clade A could be delimitated by their secondary structures, having 4–7 CBCs between the helices—as shown in Figure 6. In this sense, *T. violea* showed 3–5 CBCs with respect to *T. tomaculum*; *T. eichleriana* (ECU6) exhibited 1–5 CBCs with respect to *T. tomaculum* and 1CBC with respect to *T.* sp. (ECU5).

Clade B indicates the presence of one to eight CBCs between *T.* sp. (GER) and *T. pinicola*; 1–2 CBCs with *T. asymmetrica;* and 1–3 CBCs with *T.* sp. (ECU3). Likewise, *T. pinicola* showed 1–2 CBCs with respect to *T. albida*; 1–5 CBCs with *T. asymmetrica*; and one CBC with *T. andina*. Thereby, *T. albida* presented 1–3 CBCs with respect to *T.* sp. (ECU3) and 1–2 CBCs with *T. andina*. Finally, there were 1–4 CBCs between *T. eichleraina aff.* (ECU4) and *T. asymmetrica*. Consequently, CBCs were generally informative for distinguishing species within *Tulasnella* groups. The CBCs analyses of *Tulasnella* were in concordance with the ITS2 consensus structures and phylogeny tree results.

## 4. Discussion

The phylogenetic results taken from the ITS2 sequence distance matrix combinations allowed us to identify the two main monophyletic clades A and B with bootstrap values, ranging from 72% to 100%, and with internal (A1, A2[I–II]) and external sub-clades (B1, B2[I–III], B3), respectively. The CBCs in the ITS2 secondary structures correlated with seven different *Tulasnella* species (A1, A2, B1, B2-I, B2-II, B2-III, and B3) and three types of domains. Although this integration approach improved the inference of evolutionary relationships and the species delimitation of *Tulasnella* sequences compared to traditional approaches, which have shown discrepancies between molecular and taxonomic data [49], it is still not sufficient for defining more complex group—for example, the cryptic species *T.* sp. ECU 5 (A2-II) and *T.* sp. ECU 3 (B2-IV), and *T.* *tomaculum* (A2-I) and *T. albida* (B2-I), which remained unclassified—as also discussed by Cruz et al., 2016. [49]

*Tulasnellaceae* species are especially difficult to classify taxonomically because of their ambiguous morphological and molecular characteristics [30]. Members of the genus *Tulasnella* were studied morphologically by [50] on samples collected in Ecuador, Germany, and other countries, with some of them distinguished as *T. eichleriana*, *T.* cf. *pinicola*, *T.* sp., and *T. violea* based on the size and shape of their hyphae, basidia, sterigmata, and basidiospores. However, the morphological structures of *Tulasnella* often overlap in shape and size among species, making correct identification difficult [49].

Cruz et al. [7,49] proposed a new threshold for defining phylogenetic species in fungi—particularly *Tulasnella*. They examined the variation within species and divergence among species by using the ITS-5.8S region and the morphological data of the basidiomata of *Tulasnella* spp. Their analysis suggested that morphological characteristics alone are insufficient to delimit species of *Tulasnella—*a situation that is widespread in the Basidiomycota. Furthermore, in the phylogenetic tree provided by Cruz et al., 2014 [7] the specimen DC271 was classified within the subclade *Tulasnella* sp. ECU4—which, according to the morphology sequences, belongs to the clade *T. asymmetrica*. By contrast, our phylogenetic tree identified the specimen DC271 as the species most related to *T. eichleriana* (B2-III) and *T.* *asymmetrica*; in agreement with Cruz et al., 2014, [7] their clades were related to each other morphologically by the similar growth of their anamorphs in culture.

Consequently, motivated by providing better solutions than single-and multi-locus strategies for species delimitation, we applied a new phylogenetic approach based on distance matrix combination.

The Distatis tree results shown in Figure 5 indicate two delimited clades A and B, specified according to their morphology. Clade A included subgroups (A1 and A2) that correspond to morphologically close species in *Tulasnella (T. violea* and *T. eichleriana*), with globose-to-subglobose basidiospores from Germany (DC), England (KM), Ecuador (DC) and Wales (KM)—already discussed in depth by Cruz et al., 2016 [49].

The *Tulasnella eichleriana* ECU6 and *Tulasnella eichleriana* ECU4 were externally divided into Clade A2 and Clade B2–III, respectively. This separation could be associated with the sample collection location—for example, clade A2 contained sequences sampled from Germany, England, and Ecuador (ECU6), while clade B2-III contained sequences sampled only from Ecuador (ECU4). However, we can observe some incongruences in clade A2 (*T. eichleriana*), which was internally subdivided into *T. tomaculum* from England and Wales (A2-I, in light green) and *T.* sp. from Ecuador-ECU5 (A2-II, in light green).

On the other hand, clade B contained subgroups (B1, B2, and B3); B1 included *T.* spp. and *Uncultured Tulasnella* from Germany and Ecuador, and their sequences showed a high bootstrap value (100% BP) in the taxonomy. Clade B2 was subdivided into four (I–IV) classes: *T. pinicola* (Germany and Ecuador), *T. albida* (England and Wales), *T. asymmetrica* (Australia), and *T.* sp. *(ECU3)*, with BP values between 72% and 99%. In agreement with Cruz et al.’s phylogenetic results, all these were clustered into the same clade B2.

Finally, clade B3 (*T. andina* from Ecuador) was not described morphologically by Cruz et al. [49]. However, its monophyletic clade is supported by a high bootstrap value (100%); and in accordance with the molecular results [49], this clade is near to the *T. asymmetrica*, *T. albida*, *T*. spp. (ECU3), and *T. pinicola* clade B2-I.

Although the distance method has been successfully tested, some inconsistencies were still identified in our phylogenetic tree (Figure 5). These incongruences were specifically identified in clade A2[I-II] (*T. tomaculum*, *T.* spp. ECU5) and in clade B2-IV (*T.* spp. ECU3), which were misclassified in the tree; this can be attributed to their variability and morphologic traits, as has been mentioned by Cruz et al. [49].

This information was also integrated with secondary structure modelling and CBC identification between helices to improve *Tulasnella* species delimitation.

The *Tulasnella* ITS2 models in this work did not match the consensus models reported in the literature [50,51], which normally have four stems (helices)—with the longest being stem III—and a UGGU motif. Stem II contains a pyrimidine–pyrimidine bulge and the loop between the stems has a pronounced adenine—both of which are common features of ITS2 among angiosperms. The species of the *Tulasnella* group have truly divergent ITS-5.8S regions ranging from 600 to 900 bp for the different species, allowing for the formation of ingroups, as reported by Cruz et al. [7,49] and Freitas et al. [52]. The presence of indels in this group represents interspecific variability, displaying different consensus secondary structure prototypes and revealing three types of models.

Some molecular phylogenetic studies on the nrITS-5.8S sequences of *Tulasnella* species previously isolated from the mycorrhizas of epiphytic orchids have shown genomic variability among clones that is difficult to interpret as intra- or interspecific variations or to correlate with described *Tulasnella* species; thus, it could be attributed to the apparent variability in the number of helices and structural details—for example, four and six helices were modelled in this study.

Due to the diversity of mycorrhizal *Tulasnella* associated with epiphytic orchids, they show morphological variability.

In Eukaryotics that also have a predefined pattern of four helices, the slippage of RNA polymerase during transcription may result in the production of mononucleotide repeats (“UUUU”) in the RNA sequences [52,53]. These inadvertent errors in transcription may lead to an increase in the number of detected ITS2 ribotypes [54]. Furthermore, this could be attributed to the apparent variability in the number of helices and structural details that occur in the ITS1 transcript among Eukaryotics—for example, seven helices were proposed for *Digenea* [51,55,56,57,58].

According to Cao et al. [22] and Zhang et al. [59], the site covariation (CBC)—in combination with phylogenetic comparisons—is increasingly being used as a more effective method for structure prediction. Hence, to corroborate the species delimitation revealed by the phylogeny and consensus model (Type I–IV with a total of four to six domains), a minimum of nine CBCs among individual structural helices and a total of 645 CBCs were identified between the internal and external subclasses of different species, as an additional molecular marker. It has been experimentally demonstrated that taxa separated by one CBC are totally incapable of intercrossing [24,25,60], and that it could be said that they represent separate species.

## 5. Conclusions

The proposed phylogenetic method delineated the *Tulasnella* species into two main clades (A and B), with seven monophyletic internal subclades. All predicted consensus secondary structures were essential to approximate the phylogenetic and evolutionary relationships between the organisms. The ITS2 consensus models revealed three different types of domains with four and six helices, which does not match with the consensus models reported in the literature, which normally have four helices.

Clades A and B were delimitated by one to nine CBCs in the conserved regions (helices II and III), and correlated with morphological incompatibility and intragenomic variability in the ITS2 sequences. However, the integration method depends on the data’s complexity—namely, it relies on the variability in each sequence’s group.

To the best of our knowledge, this is the first study to systematically evaluate CBCs in the predicted secondary structures for the rRNA sequences that belong to *Tulasnella* species. For future work, we propose to use the artificial intelligence method of special neural networks as a species recognition approach, as well as for secondary structure modelling and automatic CBC detection.

## Figures and Tables

**Table 1:** M-GMYC statistics obtained during the identification of monophyletic clades in the AB, AF, and Distance phylogenetic trees. The Likelihood Rate is shown in Column 3 and Column 4 refers to the number of Maximum Likelihood cluster-delimited groups with more than two individuals/sequences.

Type of Tree	Method	Result of LR Test	Number of ML Clusters
AB Tree	OPAL bootstrap	0.02277398	11
AF Tree	VRNAdist	0.4530083	9
Distance tree	Distatis R	0.01520527 *	8

* Statistical significance *p* < 0.05.

**Table 2:** CBC distribution in *Tulasnella* spp.

Evolutionary Base Pared Changes	CBC = 0	CBC > 0
**CBCs among internal sub-clades**	0	212
**CBCs among external sub-clades**	21	433
TOTAL CBCs	21	645

## Data Availability

Links for the files, sequences, trees, matrices results, and dataset analyzed during the study are available at the following links: https://drive.google.com/drive/folders/1Fs7ewUhC_LJa0kSFAWdJ7G4XXPcKerJ1?usp=sharing (accessed on 3 August 2023), for easy reading. http://purl.org/phylo/treebase/phylows/study/TB2:S30176?x-access-code=9d9bc5fb651f6b5d18b25a1ac244f4dd&format=html (accessed on 3 August 2023).

## Abbreviations

BOLD	Barcode of Life
CBC	Compensatory base change
GMYC	Generalized mixed Yule-coalescent
ITS	Internal transcribed spacers
-InL	The negative log-likelihood
LRT	Likelihood ratio test
MFE	Minimum free energy
MSA	Multiple sequence alignments
ML	Maximum likelihood
MDS	Metric multidimensional scaling
NCBI	National Center for Biotechnology Information
NJ	Neighbor Joining

## Appendix A. List of Software and Packages to *Tulasnella* Species Delimitation

**Table A1:** List of programs/packages that were used in this work.

Software	Description	Link (accessed on 1 February 2022)
Genbank	Database National Center for Biotechnology Information (NCBI), to download sequences.	http://www.ncbi.nlm.nih.gov/
ITSx v1.1.3	ITS2 region extraction	https://microbiology.se/software/itsx/
Maff-add v7	Sequences alignment	http://mafft.cbrc.jp/alignment/software
Mfold v 3.6	To infer 2D structures by a MFE (free energy minimization) parameter.	http://www.unafold.org/mfold/applications/rna-folding-form.php
RNA distance V2.6.2	Distance matrix from predicted structures was obtained with RNA distance fromVienna package.	https://www.tbi.univie.ac.at/RNA/
MEGA6.0	An integrated tool for conducting automatic and manual sequence alignment and inferring phylogenetic trees.	https://www.megasoftware.net
Clustal Omega v1.2.1	Sequences alignment	http://www.clustal.org/omega/
OPAL v2.1.3	Sequences alignment	http:/opal.cs.arizona.edu
RaxML-ng v.1.2.0	Phylogenetic tree inference.	https://github.com/amkozlov/raxml-ng
DNAdist v3.69	Used to obtain the distance matrix from Which evaluates the similarity between distance matrices Opal MSA	https://evolution.genetics.washington.edu/phylip/doc/dnadist.html
DistatisR.	The compromise matrix was used as the input data to construct the third phylogenetic tree using pvclust library with R package v 1.10	https://cran.r-project.org/web/packages/DistatisR/index.html
ITS2 database	An exhaustive dataset of internal transcribed spacer 2 sequences from NCBI GenBank, accurately reannotated.	http://its2.bioapps.biozentrum.uni-wuerzburg.de/
LocARNA V2.0.0	Software to perform structural-alignment and consensus secondary structure modelling	https://rna.informatik.uni-freiburg.de/LocARNA/Input.jsp;jsessionid=115161C5C9E9AB44CC6D3C81763A1699
4SALE tool v1.7.1	Secondary Structure Alignment	http://4sale.bioapps.biozentrum.uni-wuerzburg.de/
Figtree v1.4.4	Tree visualization	http://tree.bio.ed.ac.uk/software/figtree/
pv-clust	Hierarchical Clustering Analysis library with R package V2.2-0	https://cran.r-project.org/web/packages/pvclust/index.html
Splits-gmyc	Delimiting species and automated taxonomy library with R package V1.0-20	https://rdrr.io/rforge/splits/

## Appendix B. Compensatory Base Changes in *Tulasnella* Sequences

**Table A2:** CBCs between internal sub-clades and external sub-clades A and B, and its sequence-structure distribution.

Sub-Clades A/B	Internal and External Sub-Clades	Evolutionary Base Pared Changes in *Tulasnella* spp.		
		CBCs among Internal Sub-Clades Threshold (1–9)		CBCs among External Sub-Clades Threshold (1–10)
		CBCs = 0	CBCs > 0	CBCs > 0
**A (98%)**	**A1** ***T. violea***	A1 → A1 A1 → A2-II	A1 → A2 (4–7) A1 → A2-I (3–5)	A1 → B1 (1–7)
				A1 → B2-I (1–7)
				A1 → B2-II (1–9)
				A1 → B2-III (1–6)
				A1 → B2-V (1–5)
				A1 → B-IV (1–2)
				A1 → B3
	**A2** ***T. eichleriana*** **ECU6**	A2 → A2	A2 → A2-I (1–5) A2 → A2-II (1–1)	A2 → B1(1–7)
				A2 → B2-I (1–7)
				A2 → B2-II (1–9)
				A2 → B2-III (1–5)
				A2 → B2-V (1–4)
				A2 → B2-IV (1–2)
				A2 → B3
	**A2-I** ***T. tomaculum***	A2-I → A2-I	A2-I → A2-II(1–5) -	A2-I → B1 (1–7)
				A2-I → B2-I (1–7)
				A2-I → B2-II (1–9)
				A2-I → B2-III (1–5)
				A2-I → B2-V (1–4)
				A2-I → B2-IV (1–4)
				A2-I → B3
	**A2-II** ***T.* sp ECU5**	A2-II→ A2-II	A2-II→ A2-I (1–1)	A2-II → B1 (1–7)
				A2-II → B2-I (1–7)
				A2-II → B2-II (1–9)
				A2-II → B2-III (1–5)
				A2-II → B2-V (1–4)
				A2-II → B2-IV (1–4)
				A2-II → B3
**B (89%)**	**B1** ***T.* sp (GER)**	B1 → B1 B1 → B2-II B1 → B2-II	B1 → B2-I (1–8) B1 → B2-V (1–2) B1 → B2-IV (1–3)	
	**B2-I** ***T. pinicola***	B2-I → B2-I B2-I → B4 B2-I → B2-IV	B2-I → B2-II (1–2) B2-I → B2-V (1–5) B2-I → B2-II (1–1)	
	**B2-II** ***T. albida***	B2-II → B2-II B2-II → B2-III B2-II → B2-V	B2-II → B2-IV (1–3) B2-II → B2-II (1–2)	
	**B2-III** ***T. eichleriana* ECU4**	B2-III → B2-III B2-III → B2-IV	B2-III→B2-V (1–4) B2-III → B2-II (1–5)	
	**B2-V** ***T. asymmetrica***	B2-V → B2-V B2-V → B2-IV	B2-V→ B3(1–4)	
	**B2-IV** ***T.* sp ECU3**	B2-IV → B2-IV B2-IV→ B3		
	**B3** ***T. andina***	B3 → B3		
**Total**		**∑ CBC_i_ = 0** **21**	**∑ CBC_i_ > 0** **212**	**∑ CBC_e_ > 0** **433**

## References

[1] Chethana K.W., Manawasinghe I.S., Hurdeal V.G., Bhunjun C.S., Appadoo M.A., Gentekaki E., Raspé O., Promputtha I., Hyde K.D. (2021). What are fungal species and how to delineate them?. Fungal Divers..

[2] Ding X., Xiao J.H., Li L., Conran J.G., Li J. (2019). Congruent species delimitation of two controversial gold-thread nanmu tree species based on morphological and restriction site-associated DNA sequencing data. J. Syst. Evol..

[3] Woese C.R., Fox G.E. (1977). Phylogenetic structure of the prokaryotic domain: The primary kingdoms. Proc. Natl. Acad. Sci. USA.

[4] Stengel A., Stanke K.M., Quattrone A.C., Herr J.R. (2022). Improving Taxonomic Delimitation of Fungal Species in the Age of Genomics and Phenomics. Front. Microbiol..

[5] Frazer K.A., Elnitski L., Church D.M., Dubchak I., Hardison R.C. (2003). Cross-species sequence comparisons: A review of methods and available resources. Genome Res..

[6] DeSalle R., Goldstein P. (2019). Review and interpretation of trends in DNA barcoding. Front. Ecol. Evol..

[7] Cruz D., Suárez J.P., Kottke I., Piepenbring M. (2014). Cryptic species revealed by molecular phylogenetic analysis of sequences obtained from basidiomata of *Tulasnella*. Mycologia.

[8] Edgar R.C., Batzoglou S. (2006). Multiple sequence alignment. Curr. Opin. Struct. Biol..

[9] Agüero-Chapin G., Jiménez Y., Sánchez-Rodríguez A., Molina-Ruiz R., Vivanco O., Antunes A. (2021). DISTATIS: A Promising Framework to Integrate Distance Matrices in Molecular Phylogenetics. Curr. Top. Med. Chem..

[10] Janies D., Studer J., Handelman S., Linchangco G. (2013). A comparison of supermatrix and supertree methods for multilocus phylogenetics using organismal datasets. Cladistics.

[11] Abeysundera M., Kenney T., Field C., Gu H. (2014). Combining distance matrices on identical taxon sets for multigene analysis with singular value decomposition. PLoS ONE.

[12] Ihrmark K., Bödeker I., Cruz-Martinez K., Friberg H., Kubartova A., Schenck J., Strid Y., Stenlid J., Brandström-Durling M., Clemmensen K.E. (2012). New primers to amplify the fungal ITS2 region–evaluation by 454-sequencing of artificial and natural communities. FEMS Microbiol. Ecol..

[13] Schoch C.L., Seifert K.A., Huhndorf S., Robert V., Spouge J.L., Levesque C.A., Chen W., Consortium F.B., Fungal Barcoding Consortium, Fungal Barcoding Consortium Author List (2012). Nuclear ribosomal internal transcribed spacer (ITS) region as a universal DNA barcode marker for *Fungi*. Proc. Natl. Acad. Sci. USA.

[14] Coleman A.W. (2003). ITS2 is a double-edged tool for eukaryote evolutionary comparisons. TRENDS Genet..

[15] Coleman A.W., Vacquier V.D. (2002). Exploring the phylogenetic utility of ITS sequences for animals: A test case for abalone (Haliotis). J. Mol. Evol..

[16] Schultz J., Maisel S., Gerlach D., Müller T., Wolf M. (2005). A common core of secondary structure of the internal transcribed spacer 2 (ITS2) throughout the Eukaryota. RNA.

[17] Koetschan C., Foerster F., Keller A., Schleicher T., Ruderisch B., Schwarz R., Müller T., Wolf M., Schultz J. (2010). The ITS2 Database III—Sequences and structures for phylogeny. Nucleic Acids Res..

[18] Müller T., Philippi N., Dandekar T., Schultz J., Wolf M. (2007). Distinguishing species. RNA.

[19] Caisová L., Marin B., Melkonian M. (2013). A consensus secondary structure of ITS2 in the Chlorophyta identified by phylogenetic reconstruction. Protist.

[20] Schultz J., Wolf M. (2009). ITS2 sequence–structure analysis in phylogenetics: A how-to manual for molecular systematics. Mol. Phylogenetics Evol..

[21] Sundaresan N., Jagan E.G., Kathamuthu G., Pandi M. (2019). Internal transcribed spacer 2 (ITS2) molecular morphometric analysis based species delimitation of foliar endophytic fungi from *Aglaia elaeagnoidea*, *Flacourtia inermis* and *Premna serratifolia*. PLoS ONE.

[22] Cao R., Tong S., Luan T., Zheng H., Zhang W. (2022). Compensatory Base Changes and Varying Phylogenetic Effects on Angiosperm ITS2 Genetic Distances. Plants.

[23] Wolf M., Chen S., Song J., Ankenbrand M., Müller T. (2013). Compensatory Base Changes in ITS2 Secondary Structures Correlate with the Biological Species Concept Despite Intragenomic Variability in ITS2 Sequences—A Proof of Concept. PLoS ONE.

[24] Wolf M., Friedrich J., Dandekar T., Muller T. (2005). CBCAnalyzer: Inferring phylogenies based on compensatory base changes in RNA secondary structures. Silico Biol..

[25] Ruhl M., Wolf M., Jenkins T.M. (2009). Compensatory base changes illuminate morphologically difficult taxonomy. Mol. Phylogenetics Evol..

[26] Agüero-Chapin G., Sánchez-Rodríguez A., Hidalgo-Yanes P.I., Pérez-Castillo Y., Molina-Ruiz R., Marchal K., Vasconcelos V., Antunes A. (2011). An alignment-free approach for eukaryotic ITS2 annotation and phylogenetic inference. PLoS ONE.

[27] Zahin T., Abrar M.H., Rahman M., Tasnim T., Bayzid M.S., Rahman A. (2019). An Alignment-free Method for Phylogeny Estimation using Maximum Likelihood. bioRxiv.

[28] Zuo G. (2021). CVTree: A parallel alignment-free phylogeny and taxonomy tool based on composition vectors of genomes. Genom. Proteom. Bioinform..

[29] Cheng J., Cao F., Liu Z. (2013). AGP: A multimethods web server for alignment-free genome phylogeny. Mol. Biol. Evol..

[30] Abeysundera M., Field C., Gu H. (2011). Phylogenetic analysis based on spectral methods. Mol. Biol. Evol..

[31] Benson D.A., Karsch-Mizrachi I., Lipman D.J., Ostell J., Sayers E.W. (2009). GenBank. Nucleic Acids Res..

[32] Bengtsson-Palme J., Ryberg M., Hartmann M., Branco S., Wang Z., Godhe A., Nilsson R.H. (2013). Improved software detection and extraction of ITS1 and ITS2 from ribosomal ITS sequences of fungi and other eukaryotes for analysis of environmental sequencing data. Methods Ecol. Evol..

[33] Katoh K., Standley D.M. (2013). MAFFT multiple sequence alignment software version 7: Improvements in performance and usability. Mol. Biol. Evol..

[34] Suzuki R., Shimodaira H. (2006). Pvclust: An R package for assessing the uncertainty in hierarchical clustering. Bioinformatics.

[35] Zuker M. (2003). Mfold web server for nucleic acid folding and hybridization prediction. Nucleic Acids Res..

[36] Hofacker I.L., Fontana W., Stadler P.F., Bonhoeffer L.S., Tacker M., Schuster P. (1994). Fast folding and comparison of RNA secondary structures. Monatshefte Chem. Chem. Mon..

[37] Lorenz R., Bernhart S.H., Zu Siederdissen C.H., Tafer H., Flamm C., Stadler P.F., Hofacker I.L. (2011). ViennaRNA Package 2.0. Algorithms Mol. Biol..

[38] Tamura K., Stecher G., Peterson D., Filipski A., Kumar S. (2013). MEGA6: Molecular evolutionary genetics analysis version 6.0. Mol. Biol. Evol..

[39] Sievers F., Wilm A., Dineen D., Gibson T.J., Karplus K., Li W., Lopez R., McWilliam H., Remmert M., Söding J. (2011). Fast, scalable generation of high-quality protein multiple sequence alignments using Clustal Omega. Mol. Syst. Biol..

[40] Anderson C., Strope C., Moriyama E. (2011). Assessing Multiple Sequence Alignments Using Visual Tools. Bioinformatics: Trends and Methodologies.

[41] Wheeler T.J., Kececioglu J.D. (2007). Multiple alignment by aligning alignments. Bioinformatics.

[42] Stamatakis A. (2014). RAxML version 8: A tool for phylogenetic analysis and post-analysis of large phylogenies. Bioinformatics.

[43] Fujisawa T., Barraclough T.G. (2013). Delimiting species using single-locus data and the Generalized Mixed Yule Coalescent approach: A revised method and evaluation on simulated data sets. Syst. Biol..

[44] Ankenbrand M.J., Keller A., Wolf M., Schultz J., Förster F. (2015). ITS2 database V: Twice as much. Mol. Biol. Evol..

[45] Liao M.L., Dong Y.W., Somero G.N. (2021). Thermal adaptation of mRNA secondary structure: Stability versus lability. Proc. Natl. Acad. Sci. USA.

[46] Will S., Joshi T., Hofacker I.L., Stadler P., Backofen R. (2012). LocARNA-P: Accurate boundary prediction and improved detection of structural RNAs. Rna.

[47] Li M., Zhao H., Zhao F., Jiang L., Peng H., Zhang W., Simmons M.P. (2019). Alternative analyses of compensatory base changes in an ITS2 phylogeny of Corydalis (Papaveraceae). Ann. Bot..

[48] Seibel P., Muller T., Dandekar T., Schultz J., Wolf M. (2006). 4SALE—a tool for synchronous RNA sequence and secondary structure alignment and editing. BMC Bioinform..

[49] Cruz D., Suárez J.P., Piepenbring M. (2016). Morphological revision of Tulasnellaceae, with two new species of *Tulasnella* and new records of *Tulasnella* spp. for Ecuador. Nova Hedwig.

[50] Roberts P. (1992). Spiral-spored *Tulasnella* species from Devon and the New Forest. Mycol. Res..

[51] Keller A., Förster F., Müller T., Dandekar T., Schultz J., Wolf M. (2010). Including RNA secondary structures improves accuracy and robustness in reconstruction of phylogenetic trees. Biol. Direct.

[52] Freitas E.F.S., da Silva M., Cruz E.D.S., Mangaravite E., Bocayuva M.F., Veloso T.G.R., Selosse M.-A., Kasuya M.C.M. (2020). Diversity of mycorrhizal *Tulasnella* associated with epiphytic and rupicolous orchids from the Brazilian Atlantic Forest, including four new species. Sci. Rep..

[53] Schulenburg J., Englisch U., Wägele J.W. (1999). Evolution of ITS1 rDNA in the Digenea (Platyhelminthes: Trematoda): 3′ end sequence conservation and its phylogenetic utility. J. Mol. Evol..

[54] Coleman A.W. (2007). Pan-eukaryote ITS2 homologies revealed by RNA secondary structure. Nucleic Acids Res..

[55] Levinson G., Gutman G.A. (1987). Slipped-strand mispairing: A major mechanism for DNA sequence evolution. Mol. Biol. Evol..

[56] Hillis D.M., Dixon M.J. (1991). Ribosomal DNA: Molecular evolution and phylogenetic inference. Q. Rev. Biol..

[57] Rampersad S.N., Hosein F.N., Carrington C.V.F. (2014). Sequence exploration reveals information bias among molecular markers used in phylogenetic reconstruction for *Colletotrichum* species. SpringerPlus.

[58] Rampersad S.N. (2014). ITS1, 5.8 S and ITS2 secondary structure modelling for intra-specific differentiation among species of the *Colletotrichum gloeosporioides* sensu lato species complex. SpringerPlus.

[59] Zhang W., Tian W., Gao Z., Wang G., Zhao H. (2020). Phylogenetic utility of rRNA ITS2 sequence-structure under functional constraint. Int. J. Mol. Sci..

[60] Réblová M., Réblová K., Štěpánek V. (2015). Molecular systematics of *Barbatosphaeria* (*Sordariomycetes*): Multigene phylogeny and secondary ITS structure. Persoonia-Mol. Phylogeny Evol. Fungi.

